# Periodic Visual Stimulation Induces Resting-State Brain Network Reconfiguration

**DOI:** 10.3389/fncom.2018.00021

**Published:** 2018-03-28

**Authors:** Daqing Guo, Fengru Guo, Yangsong Zhang, Fali Li, Yang Xia, Peng Xu, Dezhong Yao

**Affiliations:** ^1^MOE Key Lab for Neuroinformation, The Clinical Hospital of Chengdu Brain Science Institute, University of Electronic Science and Technology of China, Chengdu, China; ^2^School of Computer Science and Technology, Southwest University of Science and Technology, Mianyang, China

**Keywords:** periodic visual stimulus, steady-state visual evoked potentials (SSVEP), network reconfiguration, graph theoretical analysis, brain network, functional connectivity, EEG

## Abstract

Periodic visual stimulation can evoke the steady-state visual potential (SSVEP) in the brain. Owing to its superior characteristics, the SSVEP has been widely used in neural engineering and cognitive neuroscience studies. However, the underlying mechanisms of the SSVEP are not well understood. In this study, we introduced a brain reconfiguration methodology to explore the possible mechanisms of the SSVEP. The EEG data from five periodic stimuli consistently indicated that the periodic visual stimulation could induce resting-state brain network reconfiguration and that the responses evoked by the stimuli were correlated to the network reconfiguration indexes. For each stimulus frequency, larger response amplitudes corresponded to higher reconfiguration indexes from the resting-state network to a stimulus-evoked network. These findings demonstrate that an external periodic visual stimulation can induce the modification of intrinsic oscillatory activities by reconfiguring resting-state activity at a network level, which could facilitate the responses evoked by the stimulus. These findings provide new insights into the response mechanisms of periodic visual stimulation.

## 1. Introduction

Rhythmic brain activity is a key mechanism in information transmission within and between brain circuits, and plays an important role in neural processing and behavior (Engel et al., 2001; Thut et al., 2012). This activity can be modulated by external brain stimulation approaches, which could provide a paradigm to manipulate the intrinsic oscillatory properties of driven networks in a controlled manner via an input-driven mechanism (Ozer et al., 2009; Uzuntarla et al., 2012, 2015; Herrmann et al., 2016; Guo et al., 2017; Grossman et al., 2017). To date, several typical noninvasive stimulation methods, i.e., deep brain stimulation (DBS), transcranial magnetic stimulation (TMS), transcranial alternating current stimulation (tACS), and periodic visual stimulation, have been shown to shape brain activity; these methods have received increasing interest in recent studies and are utilized increasingly in basic and clinical research (Alagapan et al., 2016; Ruhnau et al., 2016; Kelley et al., 2018). These brain stimulation methods could generate the selective engagement of endogenous (intrinsic) oscillations, effectively allowing them to serve as potential means of manipulating and controlling cognition and treating neurobiological disorders (Fox et al., 2014; Helfrich et al., 2014; Parkin et al., 2015).

Among these stimulation methods, periodic visual stimulation is non-invasive and can probe frequency-specific brain activity, allowing wide use in cognitive neuroscience, neural engineering, and clinical studies (Vialatte et al., 2010; Zhang et al., 2014; Zhang Y. et al., 2016; Zhang Y.S. et al., 2016, 2017; Sharon and Nir, 2017). This method can evoke robust components that have the same fundamental frequency of the stimulus as well as its harmonics. This kind of stimulation usually serves as a frequency tag or an encoder for various user commands. For example, in the BCI field, the BCI paradigms based on periodic visual stimulation have received increasing attentions (Wang et al., 2016; Jiao et al., 2017; Zhang Y. et al., 2017), and the applications include wheelchair control (Li et al., 2013), BCI spellers (Li et al., 2016), detecting number processing and mental calculation in patients (Li et al., 2015), etc. In addition to adopting it as a research tool, research communities are also interested in the brain mechanism underlying the evoked responses to periodic visual stimulation (Thorpe et al., 2007; Capilla et al., 2011; Roberts and Robinson, 2012). These studies have included assessments of the source location (Srinivasan et al., 2006) and the evoked response generation mechanisms (Capilla et al., 2011) and computational modeling simulations (Roberts and Robinson, 2012; Herrmann et al., 2016). In our previous studies, we explored the mechanisms of the SSVEP from the aspect of brain networks. We found that the stimulus-evoked brain network topological properties were significantly correlated with the evoked responses (Zhang et al., 2013b, 2015). In another study, with a resting-state dataset, we found a significant association between evoked responses and resting-state brain network topology at stimulus frequencies (Zhang et al., 2013a).

There is accumulating evidence that the resting state may provide a window to understand cognition and neurobiological disorders of the brain. This type of advantage has made resting-state brain networks a hot topic in current neuroscience studies. During the resting state, no task is performed, but the brain shows a level of spontaneous activity that reflect the potential processing abilities of neural systems (Raichle et al., 2001; Deco et al., 2013). Studies indicate that whole-brain resting-state network architecture provides a basis for task-evoked network architecture (Cole et al., 2014). Interestingly, the task performance of a subject can be predicted by resting-state brain network properties or the reconfiguration from the resting-state brain network to the task-state brain network (Schultz and Cole, 2016). In recent years, discovering the dynamic reorganization of functional brain networks has received increasing attention, and studies indicate that cognitive behaviors are related to the reconfiguration of brain network architecture (Bassett et al., 2011; Krienen et al., 2014; Braun et al., 2015; Shine et al., 2016; Finc et al., 2017; Hearne et al., 2017). For instance, in a cognitive reasoning task (Hearne et al., 2017), task engagement was accompanied by a significant reconfiguration in functional brain modules, and increasing reasoning complexity led to a merging of resting-state modules. In addition, higher reasoning accuracy was associated with larger increases in global network efficiency within the reconfigured task modules.

Inspired by these works on brain reconfigurations, here, we explored the effect of the reconfigurations between resting-state and stimulus-evoked networks on evoked responses. In the current study, we tested the hypothesis that the updates from the resting state to stimulus-evoked state could facilitate the generation of the responses. According to the previous two studies using network analysis (Zhang et al., 2013a,b), we expected that larger brain network reconfigurations would benefit larger responses.

## 2. Materials and methods

### 2.1. EEG datasets

The datasets were collected from 21 healthy subjects with normal or corrected-to-normal vision. Prior to the experiment, the purpose and procedure of the experiment were explained to each subject, and each subject was asked to read and sign an informed consent. The study was approved by the Human Research and Ethics Committee at the University of Electronic Science and Technology of China. EEG data were collected with 64 Ag-AgCl electrodes using an extended 10–20 system (Brain Products GmbH, Germany), and sampled at 1,000 Hz with an 0.01–100 Hz bandpass filter and a 50 Hz notch filter. The reference electrode was FCz, and the ground electrode was AFz. The impedance was kept below 10 kΩ for all electrodes during the experiment. Horizontal and vertical electrooculograms (EOGs) were simultaneously recorded during the experiment.

During the experiment, 2 min of eyes-closed resting-state data were first collected for each subject. In each following session, one frequency was randomly selected from the frequency set (7.5, 12, 15, 20, and 30 Hz) to generate the periodic visual stimulus to collect SSVEP data for 1 min. The frequency sequence was randomized across subjects. There was a rest period of 2–3 min between two successive sessions. The visual stimulus was displayed on a laptop with a 13-inch monitor with a refresh rate of 60 Hz. The subjects were seated in a comfortable armchair approximately 60 cm away from the center of the monitor. Subjects were requested to gaze binocularly at each flickering stimulus.

### 2.2. Data processing

#### 2.2.1. Data preprocessing

Two channels, i.e., TP9 and TP10 were discarded in the subsequent analysis due to insufficient contact with the scalp and excessive artifact. All EEG data were bandpass filtered between 1 and 100 Hz, and then resampled to 250 Hz. For each subject, the first seven non-overlapping artifact-free 10-s-long epochs were selected from the resting-state data, and the first three to five non-overlapping and artifact-free 4-s-long epochs were selected from the periodic stimulation data. The criteria for the data to be artifact-free were that the data did not include signals from eye blinks, eye movements or muscle activity and the signal amplitude did not exceed 100 μV. After obtaining the data epochs, all data were re-referenced to the zero reference using the reference electrode standardization technique (REST) (http://www.neuro.uestc.edu.cn/rest/; Yao, 2001).

#### 2.2.2. SSVEP data processing

To measure the brain responses to the periodic stimulation, we directly calculated the amplitude of the frequency using a fast Fourier transform (FFT) (Srinivasan et al., 2006). To eliminate the possible effect of backgrounds across subjects, we expressed the evoked responses as the signal-to-noise ratios (SNRs) (Zhang et al., 2013b). The SNR was computed as the ratio of the power at the stimulus frequency [*P*(*f*)] divided by the mean power of the 1 Hz band centered on the stimulus frequency but excluding the stimulus frequency itself, as defined in formula (1).

(1) $$SNR=\frac{P(f)}{\frac{1}{10}\sum_{i=1}^{5}\left[P(f-0.1\times i)+P(f+0.1\times i)\right]}$$

For each stimulus frequency, the SNRs for the nine electrodes (P3, Pz, P4, PO3, POz, PO4, O1, Oz, O2) were calculated in each epoch. Then, these SNRs were further averaged across the epochs and electrodes to yield the final SNRs for each subject as the evoked responses.

#### 2.2.3. Resting-state and stimulus-evoked networks construction

Because we wanted to investigate the reconfigurations between the resting-state and stimulus-evoked networks, we calculated the brain networks under these two conditions at the same frequencies. For the five stimulus frequencies, we calculated five frequency-specific networks for resting state data and stimulus-evoked data independently. The volume conduction can influence EEG network construction, so to reduce this effect, we only chose nineteen standard electrodes, i.e., Fp1, Fp2, F7, F3, Fz, F4, F8, T7, C3, Cz, C4, T8, P7, P3, Pz, P4, P8, O1, and O2, as the nodes used to construct the networks. Based on the aim of creating a network at a specific frequency, the coherence was utilized to measure the functional connectivity between pairs of electrodes. Coherence represents the linear relationship at a specific frequency between two signals x(t) and y(t), which is defined as (Nunez et al., 1997):

(2) $$C(f)=\frac{{\left|C_{xy}(f)\right|}^{2}}{C_{xx}(f)C_{yy}(f)}$$

where *C*_*xy*_(*f*) is the cross-spectrum between *x*(*t*) and *y*(*t*), and *C*_*xx*_(*f*) and *C*_*yy*_(*f*) are the respective auto-spectra.

For each subject, five coherence matrices corresponding to the five frequencies could be calculated for each data epoch under the resting-state condition. Similarly, five coherence matrices could be calculated for each data epoch under the visual stimulation condition. For each condition, the coherence matrices of each frequency were averaged across epochs to yield the brain network connectivity matrices for subsequent analyses.

#### 2.2.4. Networks topology measurements

In this study, several network measurements were used to measure the network topology properties. The first measurement was the mean functional connectivity, which is defined as the mean connectivity strength between all the connected pairs of electrodes in each network. Other measurements were the four topological properties, i.e., clustering coefficient, characteristic path length, global efficiency, and local efficiency. In this study, we focused on the weighted network obtained with coherence.

In weighted networks, the weights indicate the connection strength and reflect a difference in the capacity and intensity of the connections between nodes. Thus, they may be a more valid approach for brain network modeling. Furthermore, using weighted networks is useful in reducing the influence of weak and potentially non-significant connections (Zhang et al., 2011). In the following section, we simply describe the formulas used to calculate the four properties. In a weighted network (*N*-by-*N*), the clustering coefficient is calculated as follows (Watts and Strogatz, 1998; Onnela et al., 2005):

(3) $$C=\frac{1}{N}\sum_{i\in N}\frac{\sum_{i,h\in N}{\left(w_{i,j}w_{i,h}w_{j,h}\right)}^{1/3}}{k_{i}(k_{i}-1)}$$

where **w**_*ij*_ is the weight between nodes *i* and *j* in the network, and **k**_*i*_ is the degree of node *i*.

When **L**_*ij*_ is denoted as the shortest path length between two nodes, then the characteristic path length of a network is calculated as follows (Newman, 2003):

(4) $$L=\frac{1}{\frac{1}{N(N-1)}\sum_{i=1}^{N}\sum_{i\neq j}^{N}1/L_{i,j}}$$

The global efficiency is computed as (Latora and Marchiori, 2001):

(5) $$E_{global}=\frac{1}{N(N-1)}\sum_{i=1}^{N}\sum_{i\neq j}^{N}1/L_{i,j}$$

With the formula 5 above, we can also compute the local efficiency of node *i* as the following:

(6) $$E_{i-local}=\frac{1}{N_{G_{i}}}\sum_{i\in G_{i}}^{N_{G_{i}}}E_{global}G_{i}$$

where *N*_*G*_*i*__ is the number of nodes in *G*_*i*_, and *G*_*i*_ denotes the subgraph composed of the set of nodes that are the direct neighbors of node *i* (Latora and Marchiori, 2001; Achard and Bullmore, 2007). Then, the local efficiency of netwrok *G* is the average of the local efficiencies of all nodes in graph *G*,

(7) $$E_{local}=\frac{1}{N}\sum_{i\in G}^{N}E_{i-local}(G_{i})$$

These topological properties were calculated by using the Brain Connectivity Toolbox (Rubinov and Sporns, 2010). More details on the descriptions of the network topology properties could be found in the reference (Rubinov and Sporns, 2010).

#### 2.2.5. Network reconfiguration measurement

To measure the network reconfiguration from the resting state to the stimulus-evoked state, we first used the Riemannian distance to calculate the distance between the resting-state network and the stimulus-evoked network. The networks are covariance matrices that belong to a smooth Riemannian manifold of symmetric positive definite (SPD) matrices (Barachant et al., 2012; Xie et al., 2017). Riemannian distance that takes into account the space is more appropriate than correlation analysis in an Euclidean space. The Riemannian distance between two networks, such *C*_1_ and *C*_2_, is defined as (Kalunga et al., 2016):

(8) $$\beta (C_{1},C_{2})={\left(\sum_{i=1}^{N}log^{2}\lambda_{i}\right)}^{1/2}$$

where λ_*i*_, *i* = 1, 2, …, *N* are the eigenvalues of ${C_{1}}^{-1}C_{2}$.

In addition, we defined two other metrics to evaluate the network reconfigurations between the resting-state network and stimulus-evoked network, i.e., the difference in the mean functional connectivity of two kinds of networks (mean connectivity alteration) and the differences in the four topological properties.

In the present study, we investigated the possible relationships between the evoked SSVEP responses and the network reconfiguration measurements by conducting across-subject Pearson's correlation analysis between the SNRs and each reconfiguration metric.

## 3. Results

### 3.1. The brain network reconfiguration driven by the periodic stimulation

**Figure 1:**
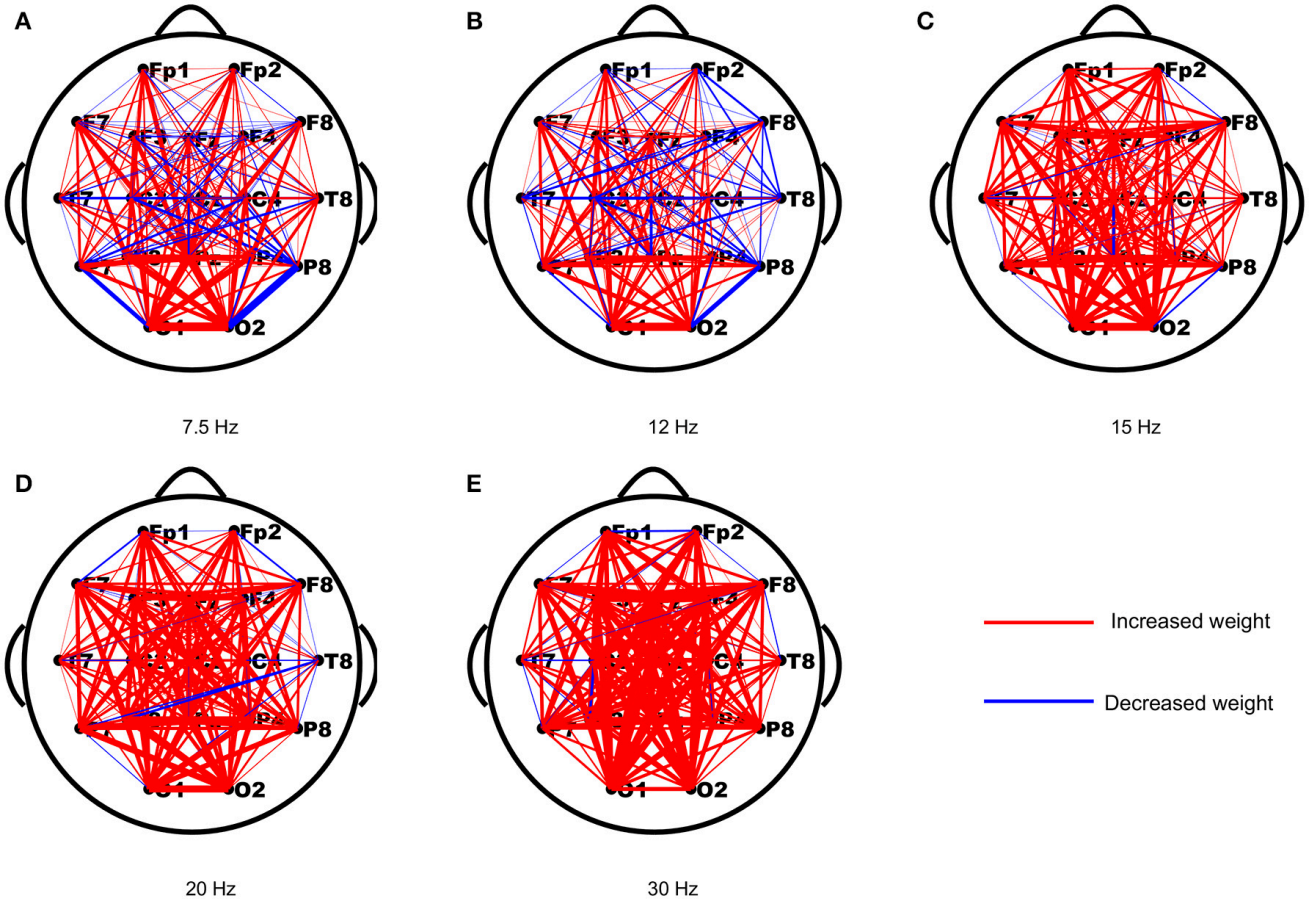
The averaged changes in functional connection weights across subjects between the resting-state and stimulus-evoked networks for the five stimulus frequencies. **(A)** 7.5 Hz; **(B)** 12 Hz; **(C)** 15 Hz; **(D)** 20 Hz; **(E)** 30 Hz. The red lines indicate increased weights and the blue lines indicate decreased weights.

**Figure 2:**
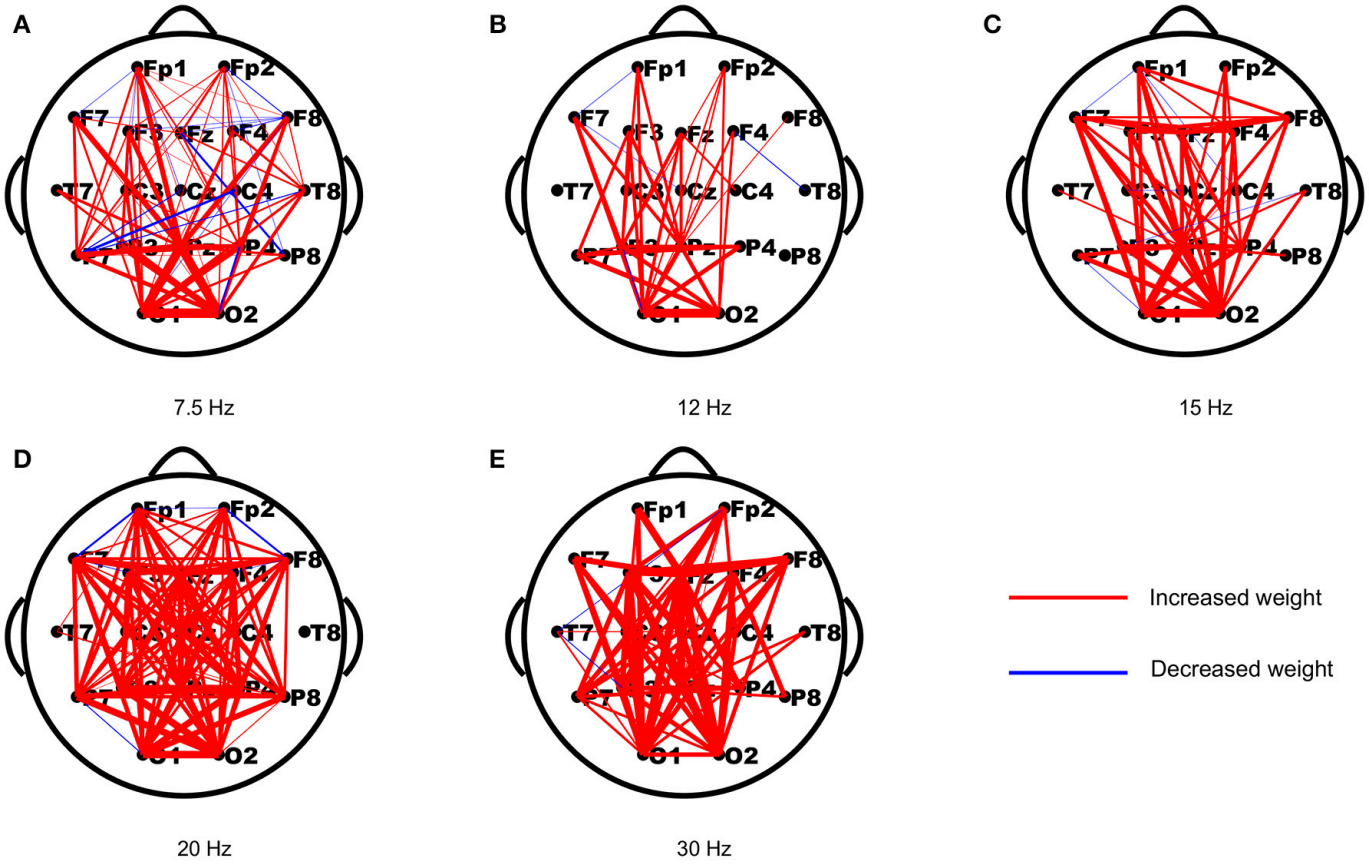
The reorganized connections that showed significant correlations with the SNRs for the five stimulus frequencies. The red and blue lines indicate the connections with increased and decreased weights (*p* < 0.05), respectively. **(A)** 7.5 Hz; **(B)** 12 Hz; **(C)** 15 Hz; **(D)** 20 Hz; **(E)** 30 Hz.

As periodic stimulation can alter the intrinsic oscillatory properties of driven networks, we first assessed the changes in functional connection strengths from the resting-state to the stimulus-evoked state induced by the stimulus. The strength differences of each connection in both networks were computed first, and then the differences of each connection were averaged across subjects to yield a network topology of weighted updates. As shown in Figure 1, most of the weights in the stimulus-evoked networks were increased compared to those in the resting-state networks, and there were fewer decreased weights in the high frequencies than in the low frequencies. Furthermore, we explored the connections that showed significant correlations with SNRs for each stimulus frequency. As seen in Figure 2, we found that the main connections existed between the parietal–occipital and frontal regions, and a small fraction of these connections showed decreased weights. These patterns may be consistent with the fact that the main sources of the SSVEP are the parietal–occipital and frontal regions (Vialatte et al., 2010), and main connectivities exist between them (Zhang et al., 2013a,b, 2015).

### 3.2. The variability in SSVEP responses across subjects

**Figure 3:**
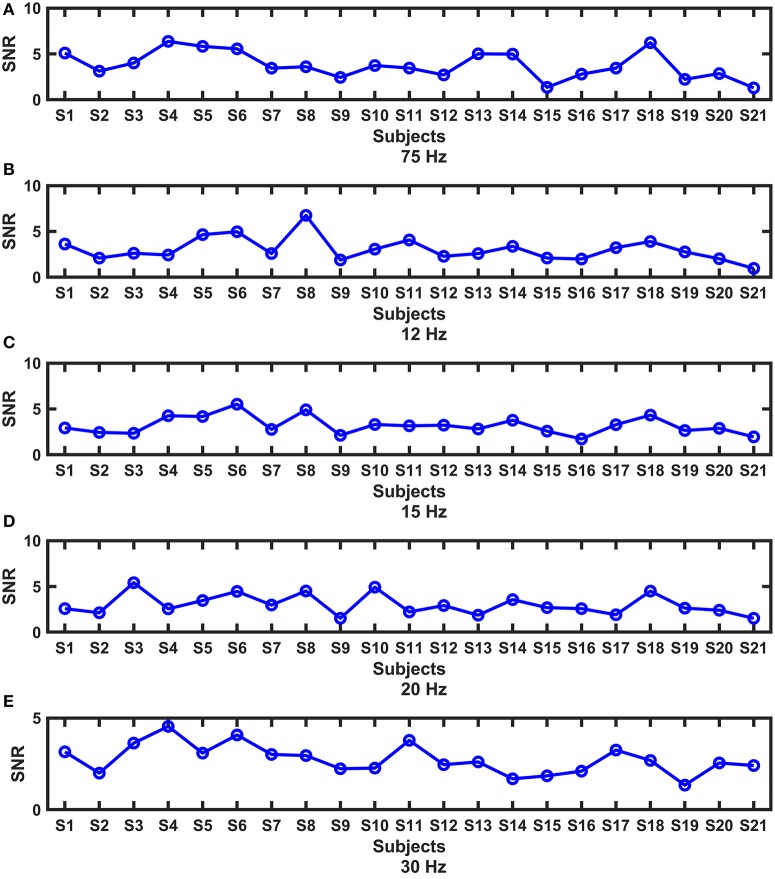
The intersubject variability of the SNRs at each frequency. **(A)** 7.5 Hz; **(B)** 12 Hz; **(C)** 15 Hz; **(D)** 20 Hz; **(E)** 30 Hz.

Periodic stimulation can evoke SSVEP responses, which are indicated by SNRs. The SNRs of all subjects at each frequency are shown in Figure 3. We found that the responses exhibited substantial intersubject variability, and different patterns existed under different frequencies. In the subsequent analysis, we wanted to know whether the variability among subjects was related to the brain network reconfiguration, as in studies in which cognitive behaviors were related to the reconfiguration of brain network architecture (Braun et al., 2015; Schultz and Cole, 2016; Hearne et al., 2017).

### 3.3. The relationship between SNRs and the network reconfiguration measurements

**Figure 4:**
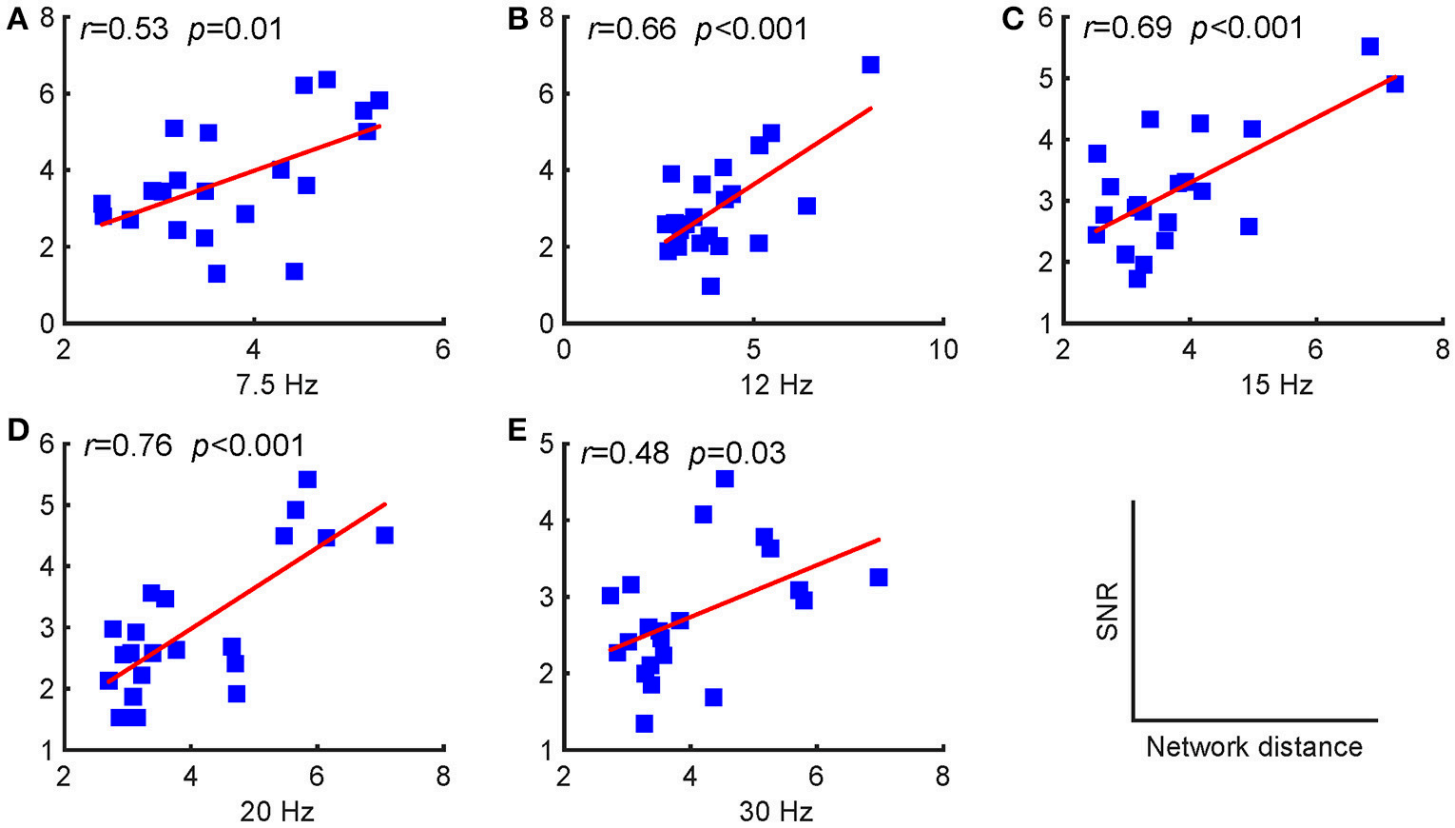
Pearson's correlations between the SNRs and the distances between the resting-state and stimulus-evoked networks for the five stimulus frequencies. **(A)** 7.5 Hz; **(B)** 12 Hz; **(C)** 15 Hz; **(D)** 20 Hz; **(E)** 30 Hz. The red lines indicate the fitted linear trend. The *r* denotes correlation coefficients, and *p* denotes the significance level of the correlation coefficients.

As stated above, periodic stimulation led to a reconfiguration between resting-state and stimulus-evoked networks. Here, we first present the network reconfiguration index as defined in the previous section to evaluate the changes for each subject and then investigate the relationships between these changes and the SNRs. We found that the SNRs of the evoked responses were positively correlated with the Riemannian distances, as shown in Figure 4. A larger distance corresponded to a relatively stronger reconfiguration between the two networks. Consequently, we can infer that larger updates between the two networks facilitate larger SSVEP responses.

**Figure 5:**
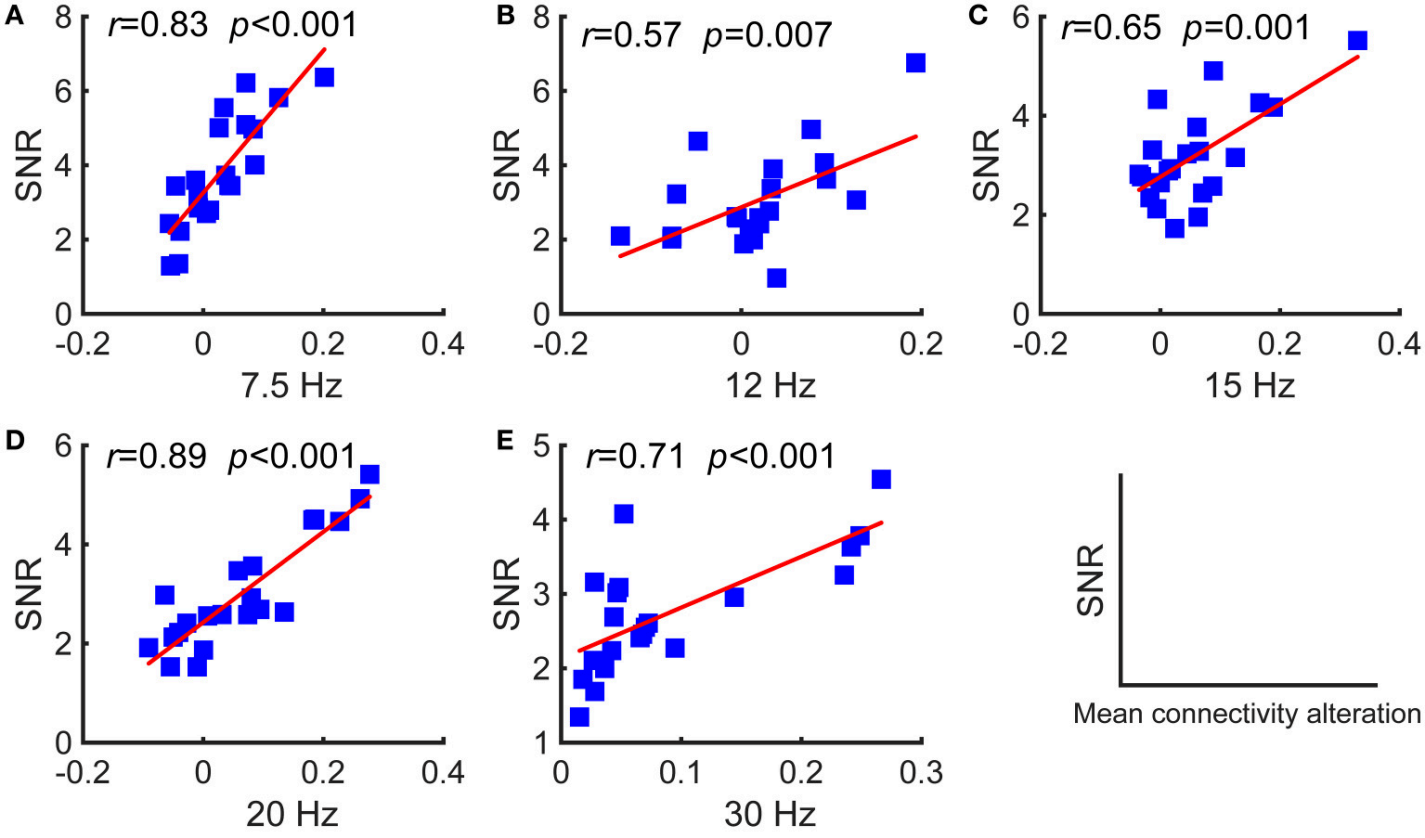
Pearson's correlations between the SNRs and the differences in the mean functional connectivity of the two types of networks for the five stimulus frequencies. **(A)** 7.5 Hz; **(B)** 12 Hz; **(C)** 15 Hz; **(D)** 20 Hz; **(E)** 30 Hz. The red lines indicate the fitted linear trend. The *r* denotes correlation coefficients, and *p* denotes the significance level of the correlation coefficients.

**Figure 6:**
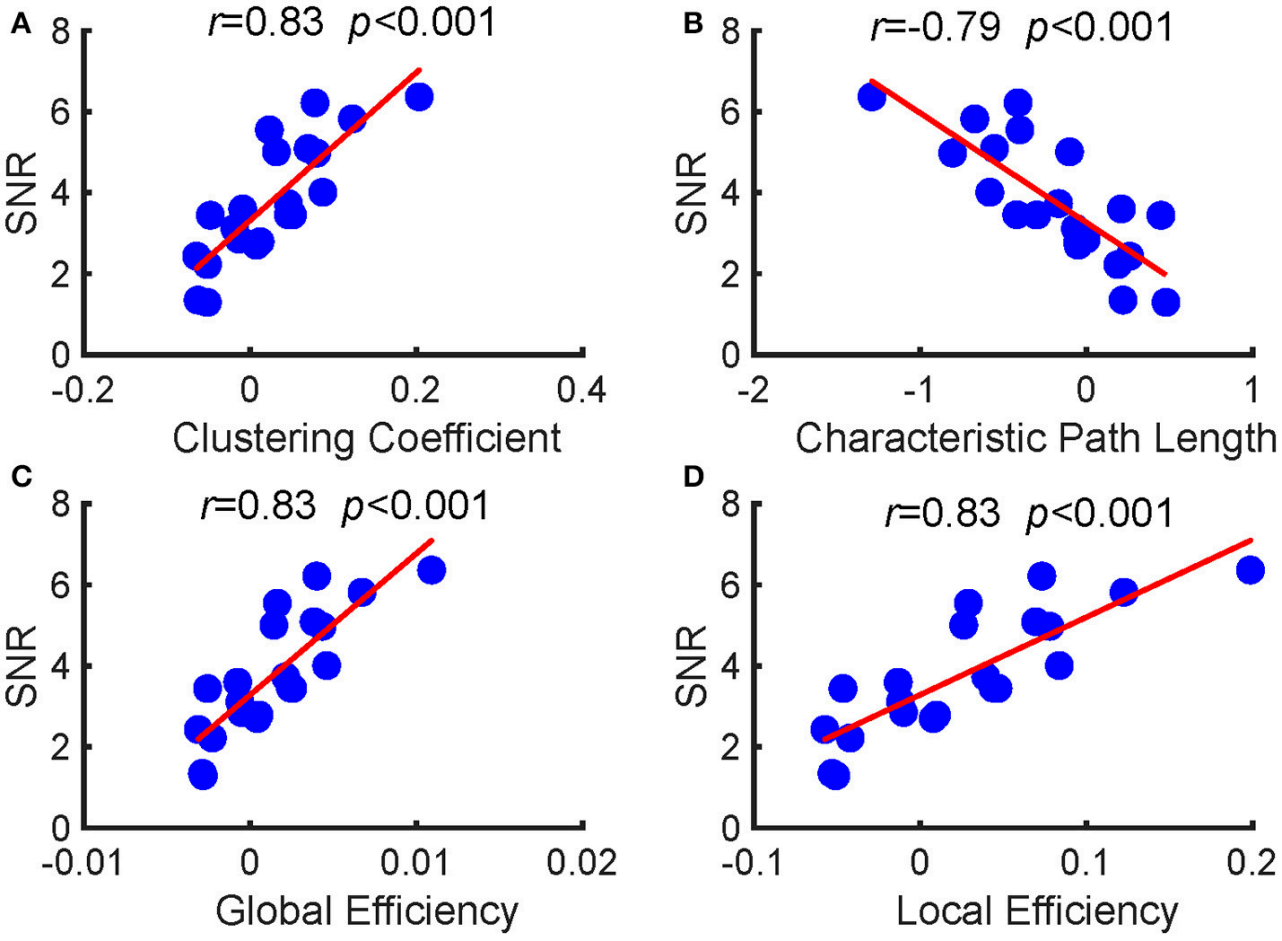
Pearson's correlations between the SNRs and the alterations in the four topological properties between the two types of networks at 7.5 Hz. **(A)** clustering coefficient; **(B)** characteristic path length; **(C)** global efficiency; **(D)** local efficiency. The red lines indicate the fitted linear trend. The *r* denotes correlation coefficients, and *p* denotes the significance level of the correlation coefficients.

Furthermore, we investigated the relationships between the SNRs and other network reconfiguration indexes. We obtained the similar results, which are as shown in Figures 5, 6 and Table 1. For each frequency, larger SSVEP SNRs corresponded to larger updates in the mean functional connectivity and the four topological properties. Therefore, the stimulus changed not only the strength of the functional connections but also the topological structure of the functional networks. Furthermore, we observed a negative correlation between the SNRs and the differences in characteristic path length, whereas there were positive correlations between the SNRs and the differences in the clustering coefficient, global efficiency, and local efficiency, as shown in Figure 6 and Table 1. Compared to the resting-state network, larger SSVEP responses corresponded to more efficient SSVEP brain network reconfigurations in the stimulus-evoked network. The results indicate that periodic visual stimulation evoked brain network reconfiguration, and the resulting more efficient brain networks indeed facilitate the corresponding SSVEP generation.

**Table 1:** The relationship between the SNRs and alterations in the four topological properties between the two types of networks at 12, 15, 20, and 30 Hz, respectively.

**Frequency (Hz)**	**Clustering coefficient**		**Characteristic path**		**Global efficiency**		**Local efficiency**	
	***r***	***p***	***r***	***p***	***r***	***p***	***r***	***p***
12	0.56	0.007	−0.64	0.002	0.56	0.007	0.56	0.008
15	0.64	0.002	−0.66	0.001	0.65	0.001	0.65	0.001
20	0.88	<0.001	−0.83	<0.001	0.89	<0.001	0.89	<0.001
30	0.71	<0.001	−0.64	0.002	0.71	<0.001	0.71	<0.001

*r denotes correlation coefficients, and p denotes the significance level of the correlation coefficients*.

## 4. Discussion

Periodic visual stimulation is a powerful tool in clinical neuroscience, cognitive neuroscience, and neural engineering (Vialatte et al., 2010). The evoked response, i.e., the SSVEP, is used as a frequency tag to study the spatial and temporal characteristics of the brain activities during a task. In recent years, the mechanisms of the SSVEP have received much attention (Thorpe et al., 2007; Zhang et al., 2013a,b, 2015; Herrmann et al., 2016), although they are not well-understood. Previous studies have indicated that adaptive reconfigurations of large-scale functional networks occur when humans are performing higher cognitive tasks (Bassett et al., 2011; Krienen et al., 2014; Braun et al., 2015; Hearne et al., 2017; Kaufmann et al., 2017). Here, we examined the reconfigurations of brain networks at specific frequencies induced by periodic visual stimulation and the relationship between the changes in brain connectivity and the responses evoked by the stimulus.

In a previous study, under the resting-state condition, the SSVEP responses of each frequency were negatively correlated with the mean functional connectivity, clustering coefficient, global efficiency, and local efficiency but positively correlated with characteristic path length (Zhang et al., 2013a). In another study, however, we found that the SSVEP responses were positively correlated with the mean functional connectivity, clustering coefficient, and global and local efficiencies but negatively correlated with the characteristic path length (Zhang et al., 2013b). These two studies separately examined the relationship between the SSVEP and the two kinds of networks. Inspired by the studies on brain network reconfiguration mentioned above, here, we investigated whether the reconfiguration of the networks constructed at a stimulus frequency occurred under the stimulus condition compared to the resting-state condition and whether the stimulus-induced network reconfiguration was associated with the evoked responses. Interestingly, there were significant changes between the intrinsic resting-state network and the stimulus-evoked network, and larger reconfigurations from the resting-state network were associated with higher evoked responses for all five stimulus frequencies. Specifically, the SSVEP responses were significantly positively correlated with the distances between the resting-state network and the stimulus-evoked network and the network reconfiguration metrics, i.e., the differences in the mean functional connectivity and the differences in the four topological properties.

In this study, no external cognitive task was performed by the subjects, but they were required to fix their attention on a flickering stimulus. Because previous studies have revealed that attention can modulate the SSVEP (Ding et al., 2006; Müller et al., 2006), we therefore infer, based on our findings, that the attended stimulus drives the adaptive reconfiguration of the network connectivity to yield robust SSVEP responses. Larger changes in functional brain networks reflect the dynamic optimization of the networks for stimulus-driven input processing and output response enhancement (Schultz and Cole, 2016). As seen in Figure 6 and Table 1, we found that the stimulus not only changed the strength of the functional connections of the frequency-specific networks but also led to the reorganization of the network's topology. The reconfiguration patterns might facilitate the global integration of information and provide a substrate for processing stimulus-driven inputs (Bola and Sabel, 2015; Alavash et al., 2016). In fact, this type of reorganization has been postulated by the global workspace theory (Dehaene and Changeux, 2011; Finc et al., 2017), and is by a number of neuroimaging studies (Palva et al., 2010; Bassett et al., 2011; Doron et al., 2012; Ekman et al., 2012; Bola and Sabel, 2015; Alavash et al., 2016).

A limitation of this work is that we only used five frequencies to investigate the mechanisms of SSVEP using the brain reconfiguration methodology. Owing to display the visual stimuli on the computer monitor using the conventional frame-based “on/off” stimulation method, we obtained small number of frequencies. We should verify the findings on large set of frequencies in our future study with LED stimulator. Another limitation of this work is that we did not adopt cognitive tasks during EEG recordings for all the subjects. For the SSVEP, it has been widely used in various cognitive tasks. In next stage, we need to validate of the results on the experiments with cognitive tasks performed by healthy subjects and patients.

The periodic visual stimulation can also evoke harmonic frequency components. In our previous studies, we found that the harmonic responses were also related to the topological properties of stimulus-evoked brain networks. It will be an interesting work to further investigate the network reconfiguration on the harmonics as the fundamental frequency. For the harmonics, we inferred that the similar relationships may exist between the harmonic responses and the corresponding network reconfiguration as those for the fundamental frequency. Besides, in current study, only one frequency was adopted in each session during the EEG recording. In some SSVEP-based BCI systems (Hwang et al., 2013), dual-frequency stimulation was used. It is necessary to carry out the experiments with double periodic visual stimulus to investigate the brain response mechanisms based on network analysis and brain reconfiguration methodology. We will carry out those researches in our future researches.

The biological implications of the main findings of this study are meaningful. First, the results shed light on new mechanisms of the SSVEP based on the reconfiguration of brain network topological architecture, which combines the resting state and stimulus-evoked state. Second, periodic visual stimulation can serve as frequency tag tool to modulate intrinsic oscillatory brain activity. Through the perspective of the brain network reconfigurations, it will be possible to further probe the mechanisms of cognition and pathological brain dynamics in cognitive and clinical investigations (Vialatte et al., 2010; Parkin et al., 2015). In recent years, investigations into the dynamics of functional connectivity patterns have received growing interest (Doron et al., 2012; Bola and Sabel, 2015; Alavash et al., 2016; Shine et al., 2016; Finc et al., 2017). Here, the static brain network was constructed based on averaged coherence connectivity matrices, which disregard the dynamic organization in functional brain networks over time. In future studies, we will investigate how the dynamic responses evoked during the stimulation period relate to dynamic changes in brain network topologies.

## 5. Conclusion

In the present study, we investigated brain network reconfigurations using periodic visual stimulation at five frequencies. The results revealed that the stimulation changed not only the strength of the functional connections but also the topological arrangements of the functional networks. The evoked SSVEP responses were significantly correlated to network reconfiguration metrics. Taken together, our findings, on one hand, can shed light on the mechanisms of the SSVEP and, on the other hand, may open new approaches to probe frequency-specific brain activity within network reconfiguration and graph theory analysis.

## Author contributions

DG, YX, PX, DY, and YZ: conceived and designed the experiments; FG, FL, and YZ: performed the experiments and data analysis; DG, FG, and YZ: wrote the paper.

### Conflict of interest statement

The authors declare that the research was conducted in the absence of any commercial or financial relationships that could be construed as a potential conflict of interest.
